# Ipsilateral Lumbosacral Dysfunction among Taxi Drivers of Left Side Steering Wheel

**Published:** 2020-02

**Authors:** Syed Asadullah ARSLAN, Gholamreza OLYAEI, Mohammad Reza HADIAN, Saeed TALIBIAN, Mir Saeed YEKANINEJAD, Kazem MALMIR, Rabiya NOOR, Muhammad Salman BASHIR

**Affiliations:** 1. Department of Physiotherapy, School of Rehabilitation, International Campus, Tehran University of Medical Sciences, Tehran, Iran; 2. Brain and Spinal Cord Injury Research Center (BASIR), Imam Khomeini Complex Hospital, Tehran University of Medical Sciences, Tehran, Iran

## Dear Editor-in-Chief

Low back pain (LBP) is a common illness that has potential sources, like intervertebral discs, facets and sacroiliac joints (SIJ). Primary source of pain in 10% to 27% of patients with mechanical LBP is SIJ which is a true diarthrodial joint; separated by a joint space containing synovial fluid and a fibrous capsule. Several mechanisms of injury may cause SIJ pain such as a direct fall on the buttocks, motor vehicle accident, and an unanticipated step into a hole or from a miscalculated height (1). SIJ is a non-weight-bearing joint that function to absorb forces from various directions. Onset of SIJ dysfunction is usually during trunk flexion when the weight of the upper trunk rotate the innominate anteriorly and downward during anterior shift and becomes fixed on the sacrum (2). Similar movement can be observed when a taxi driver gets out of the car, therefore, the aim of this study was to assess this repetitive rotational effect on the SIJ of left-hand drive taxi drivers.

After ethical approval from the concerned authority of Tehran University of Medical Sciences (TUMS), this cross-sectional study was conducted at different taxi stops of Tehran in 2018. Participants with radiation of pain towards lower limb, having postural dysfunction or limb length discrepancy, with recent history of surgery, trauma or pathological disease were excluded from the research.

Overall, 700 male taxi drivers, aged between 20–60 yr with work experience of more than one year were interviewed to fill questionnaires. Patients rate their current pain intensity from 0 (“no pain”) to 10 (“worst possible pain”) by using Numeric rating scale (NRS). The mean age, height and weight of the participants were 47.40 ± 8.90, 172.13 ± 6.05, 81.89 ± 10.77 respectively. All the participants were males and majority (96.5%) of them had right dominant hand. Most of the drivers had acquired high school (35.1%) or intermediate (45.4%) education. Only 235 (39.5%) had the habit of exercising and 248 (47.1%) used to do daily stretching exercises. Majority of the drivers, 463 (77.8%) showed no sleep disturbance and almost half of them (47.1%) were smokers.

Among 595 valid questionnaires, 219 reported LBP and were invited at physiotherapy clinic, in School of Rehabilitation to assess their SIJ. A different physiotherapist, blinded with the outcomes of the research, had performed physical assessment by applying five provocative tests for SIJ which included distraction test, thigh thrust, Flexion, Abduction and External Rotation (FABER), compression and Gaenslen’s Maneuver and studies have suggested that a battery of three or more provocation tests can predict SIJ dysfunction (1, 3).

Among 219 LBP patients, 86 were diagnosed with SIJ dysfunction by using the reference standard pain provocation tests [≥3 positive tests = SIJ-positive]. Majority of the LBP drivers, 59(26.9%) had SIJ dysfunction on left side while 27(12.3%) of them had it on right side. Mean pain intensity was 4.79 and it was significantly associated (*P*=<0.001) with SIJ dysfunction. Trauma or smaller, repetitive stresses may damage the bones, ligaments, muscles and nerves of the SIJ (4). Regarding more discomfort side of the back; 89(40.6%) reported it on right side, 59(26.9%) reported on left side while 71(32.4%) had pain on both sides. Endurance is the ability to persist achieved by repetitive contractions of muscle fibers. Repetitive contractions require a continuous supply of energy, and muscle fibers with aerobic (oxidative) capabilities (slow oxidative, fast oxidative glycolytic) are suited to the job as repetitive contractions enhance aerobic enzymes, mitochondria, and the fuels which are needed for repetitive contractions (5). Repetitive contractions of left side back muscles while getting into and out of taxi increases endurance, blood flow and nutrient supply which were insufficient in the back muscles of the right side.

Metabolites elimination and heat dissipation are essential to maintain homeostasis within muscle fibers which require sufficient blood flow. Static contractions cause marked impairment in blood flow to the muscles which might be the main explanation for muscle fatigue during isometric contractions, no matter how low the contraction (6). Therefore, both static posture and repetitive rotations should be avoided.
